# New ways to cope with depression—study protocol for a randomized controlled mixed methods trial of bouldering psychotherapy (BPT) and mental model therapy (MMT)

**DOI:** 10.1186/s13063-023-07629-x

**Published:** 2023-09-22

**Authors:** Leona Kind, Katharina Luttenberger, Vivien Leßmann, Lisa Dorscht, Christiane Mühle, Christian P. Müller, Eva-Maria Siegmann, Sophia Schneider, Johannes Kornhuber

**Affiliations:** 1 Centre for Health Services Research in Medicine, Department of Psychiatry and Psychotherapy, Friedrich-Alexander-Universität Erlangen-Nürnberg (FAU), Schwabachanlage 6, 91054 Erlangen, Germany; 2 Department of Psychiatry and Psychotherapy, Friedrich-Alexander-Universität Erlangen-Nürnberg (FAU), Schwabachanlage 6, 91054 Erlangen, Germany; 3 Centre for Drug Research, Universiti Sains Malaysia, 11800 Minden, Penang, Malaysia; 4 Institute of Psychopharmacology, Central Institute of Mental Health, Faculty of Medicine Mannheim, University of Heidelberg, Heidelberg, Germany

**Keywords:** Depression, Mental models, Exercise, Sports, Bouldering, Rock climbing, Psychotherapy, Factors of influence, Biomarkers, Osteocalcin

## Abstract

**Background:**

Due to the growing gap between the demand and supply of therapeutic services for people suffering from depression, with this study, we are investigating the effectiveness and factors of influence of new approaches in group treatments for depression. Two previous studies have already identified bouldering psychotherapy (BPT) as an effective option. It combines psychotherapeutic interventions with action- and body-oriented bouldering exercises. Mental model therapy (MMT) is a new cognitive-behavioral approach for treating depression. It focuses on identifying cognitive distortions, biases in decision making, and false assumptions and aims to correct and replace them with useful mental models. We aim to investigate the effectiveness of the interventions compared with a control group (CG) and to assess the factors of influence in a mixed methods approach.

**Methods:**

The study is being conducted as a randomized controlled intervention trial. Adult participants with unipolar depression are being randomized into three groups (BPT, MMT, or CG), and the first two groups are undergoing a 10-week treatment phase. CG follows their individual standard treatment as usual. A priori power analysis revealed that about 120 people should be included to capture a moderate effect. The primary outcome of the study is depression rated with the Montgomery and Asberg Depression Rating Scale (MADRS) before (t0), directly after (t1), and 12 months after the intervention phase (t2). Data are being collected via questionnaires, computer-assisted video interviews, and physical examinations. The primary hypotheses will be statistically analyzed by mixed model ANOVAs to compare the three groups over time. For secondary outcomes, further multivariate methods (e.g., mixed model ANOVAs and regression analyses) will be conducted. Qualitative data will be evaluated on the basis of the qualitative thematic analysis.

**Discussion:**

This study is investigating psychological and physical effects of BPT and MMT and its factors of influence on outpatients suffering from depression compared with a CG in a highly naturalistic design. The study could therefore provide insight into the modes of action of group therapy for depression and help to establish new short-term group treatments. Methodological limitations of the study might be the clinical heterogeneity of the sample and confounding effects due to simultaneous individual psychotherapy.

**Trial registration:**

ISRCTN, ISRCTN12347878. Registered 28 March 2022, https://www.isrctn.com/ISRCTN12347878.

**Supplementary Information:**

The online version contains supplementary material available at 10.1186/s13063-023-07629-x.

## Background

Depression is one of the most common mental illnesses in the world [1] and will be the most frequent health impairment by 2030 according to estimations of the World Health Organization (WHO) [2–4]. At 11.6%, the current lifetime prevalence in Germany is similar to other industrialized nations [5]. Following clinical guidelines, medication and psychotherapy are the most commonly recommended treatments for depression [6, 7]. In an outpatient care setting, psychotherapy—individual or group—is effective with moderate effect sizes [8] and almost all psychotherapies are equivalently effective [9], so patient preference plays an important role in the choice of therapy. On the other hand, more than half of the patients who receive therapy do not respond, and only one third recover [10]. During the COVID-19-crisis, the need for psychotherapeutic treatment has risen enormously due to higher prevalence rates of depression and anxiety [11]. Effective and attractive group programs might be able to respond to the lack of therapeutic resources [12], but to date, they are underrepresented in the outpatient care setting and in research [13]. They should be as effective as existing treatment options, available to more individuals, attractive, and easy to access.

To address the growing demand for treatment options, we developed two new short-term group therapies for outpatients suffering from depression: bouldering psychotherapy (BPT) and mental model therapy (MMT).

BPT is an approach that combines psychotherapeutic elements and topics (e.g., mindfulness and self-efficacy) with bouldering exercises. In recent years, therapeutic climbing, especially rock climbing and bouldering (climbing without rope in moderate heights), has moved into the focus of scientific research concerning mental health [14], resulting in several studies that have indicated positive effects of climbing and bouldering on a variety of illnesses, such as anxiety [15–17], depression [15, 18], eating disorders, post-traumatic stress disorder [16, 17], and attention deficit hyperactivity disorder [19, 20]. Such effects might be moderated by an increase in cognitive functioning [18], self-confidence, self-esteem, and self-efficacy [21], as well as affective responses [22] and social skills [18]. Up to now, there have been two randomized, controlled trials investigating the effects of a psychotherapeutic bouldering intervention, called BPT, on depressive symptoms. It was shown that BPT was superior to an active control group (CG) [23, 24] as well as to a waitlist CG directly after the end of the treatment phase [25, 26] and in the long term [27, 28]. Furthermore, BPT was not inferior to a group cognitive behavioral therapy (CBT) in its effectiveness as an intervention for depression [28] and proved to be cost-effective [29].

Mental model therapy (MMT) is based on a cognitive approach. Mental models are seen as universal rules that are applicable to private and professional life, screening for relevant information and reducing the flood of incoming information to a reasonable amount that can be processed. In contrast to CBT, which focuses on the acute emotional effects of dysfunctional thinking, MMT is based on the assumption that cognitive distortions, biases, and false assumptions have a negative influence on executive cognitive functions, such as foresight, goals, priorities, decision-making, postponing rewards, and mental flexibility in the presence of situational changes [30, 31]. Failures in such areas can result in stress, low self-esteem, and even in low social status. In a comparative analysis of different psychotherapies for depression, the good efficacy of Self-Examination Therapy stood out [32]. This therapy conveys a simple model: the resolution of changeable problems and the acceptance of the unchangeable [33]. Thus, even a single mental model can have a particularly good antidepressant effect. In MMT, we pursue the teaching of different mental models with memorable terms for cognitive distortions, thus expanding the participants’ language and conception of the world. The sources of these models lie in humanistic psychology, behavioral economics, stoic philosophy, and CBT. MMT is intended to help people develop healthy and self-actualizing personalities and thus references humanistic psychotherapy. It helps identify systematic cognitive distortions and biased decision making by using insights from behavioral economics [34]. With its acceptance of the unchangeable, MMT is in the tradition of stoic philosophy and will eventually help overcome the intention-behavior gap through the development of habits based on CBT.

In summary, MMT has been designed to have a beneficial effect in the treatment of stressful life situations and mental disorders, such as burnout and depression.

The goal of this study is threefold. First, we will compare both interventions with a naturalistic control group to test the hypothesis that individuals in the intervention groups (IGs) will experience a significantly greater reduction in their depressive symptoms than individuals in the control group (CG). CG is allowed to follow their individual prescribed treatment plans (treatment as usual) with no study-specific offer during the intervention period.

Second, we will analyze a wide range of potential mediating and moderating factors, such as anxiety [24], self-efficacy [35], mindfulness, treatment preference, and physiological factors. Moreover, we will assess blood biomarkers in an exploratory approach: Osteocalcin is a hormone produced by osteoblasts in the bone system. Besides its role in bone mineralization, it serves as a bone-brain signal. It can cross the blood-brain barrier and activate specific receptors in the brain [36, 37]. Animal studies on depression have shown sex-dependent antidepressant and anxiolytic effects of osteocalcin [38, 39]. Osteocalcin activity may be driven by physical activity in humans [40]. Furthermore, lipids, including cholesterol and low-/high-density lipoprotein, are altered in depression [41] and are influenced by patients’ physical activity [42]. Levels of sphingolipids (e.g., ceramide [43]) and corresponding enzyme activities (e.g., acid sphingomyelinase [44]) or their splice variant composition [45] are associated with major depressive disorder. Thus, serum lipid levels and the expression or activities of enzymes of the sphingolipid metabolism, including acid sphingomyelinase and sphingomyelin synthase catalyzing the reverse reaction [44], could be expected to change or normalize during BPT and MMT. These changes could be similar to the changes that occur during pharmacological antidepressant treatment [46]. Moreover, current pathomechanisms of depression include disturbances of the hypothalamic-pituitary-adrenal axis (e.g., glucocorticoid receptor sensitivity [47]), neurotrophic homeostasis (e.g., brain-derived neurotrophic factor (BDNF) levels [48]), and inflammatory processes (e.g., migration inhibitory factor (MIF) [49]). The effectiveness of BPT, MMT, or both might therefore also be reflected in the restoration of such stress regulators, growth factors, and inflammatory markers. The spectrum of biomarkers will be extended according to new literature and the detection and establishment of new assays.

In addition to the quantitative analysis, qualitative semi-structured interviews will be conducted to investigate additional specific modes of action and factors of influence.

## Methods

### Aims and hypothesis

The aim of the current study is to examine whether (a) BPT and (b) MMT are more effective in reducing participants’ depressive symptoms compared to a control group receiving treatment as usual. Therefore, we are comparing both IGs with a CG in a randomized and controlled but yet naturalistic design.

#### Research hypothesis

I. Patients in the bouldering psychotherapy (BPT) improve significantly more than patients in the control group (CG) regarding the severity of depression.
II. Patients in the mental model therapy (MMT) improve significantly more than patients in the control group (CG) regarding the severity of depression.

Effects on secondary outcomes (e.g., anxiety, self-efficacy) will be analyzed in an exploratory fashion. Regarding the biomarkers, it is expected that participation in the BPT will lead to a greater increase in blood osteocalcin and brain-derived neurotrophic factor levels than in the MMT and CG. We expect higher serum acid sphingomyelinase activities to correlate with greater improvement in depression severity in both treatment groups. Additional sphingolipid parameters, growth factors, and inflammatory markers will be analyzed and are expected to normalize in both IGs. Whereas these assumptions were based on previous studies, sample size estimation was based solely on the number of participants required for the psychotherapeutic effect and not adjusted any further.

Qualitative data will be used to explore the extent to which participants perceive the interventions to be helpful, which components or factors of influence they feel are most important, and which limitations or barriers they perceive. In line with Grawe [50], the results of the qualitative analysis will be used to further develop hypotheses on potential factors of influence with respect to BPT and MMT.

In this article, we describe the study protocol for *new ways to cope with depression*. This protocol will serve as a reference for the forthcoming papers that will report the results of the study.

### Study design and setting

**Fig. 1:**
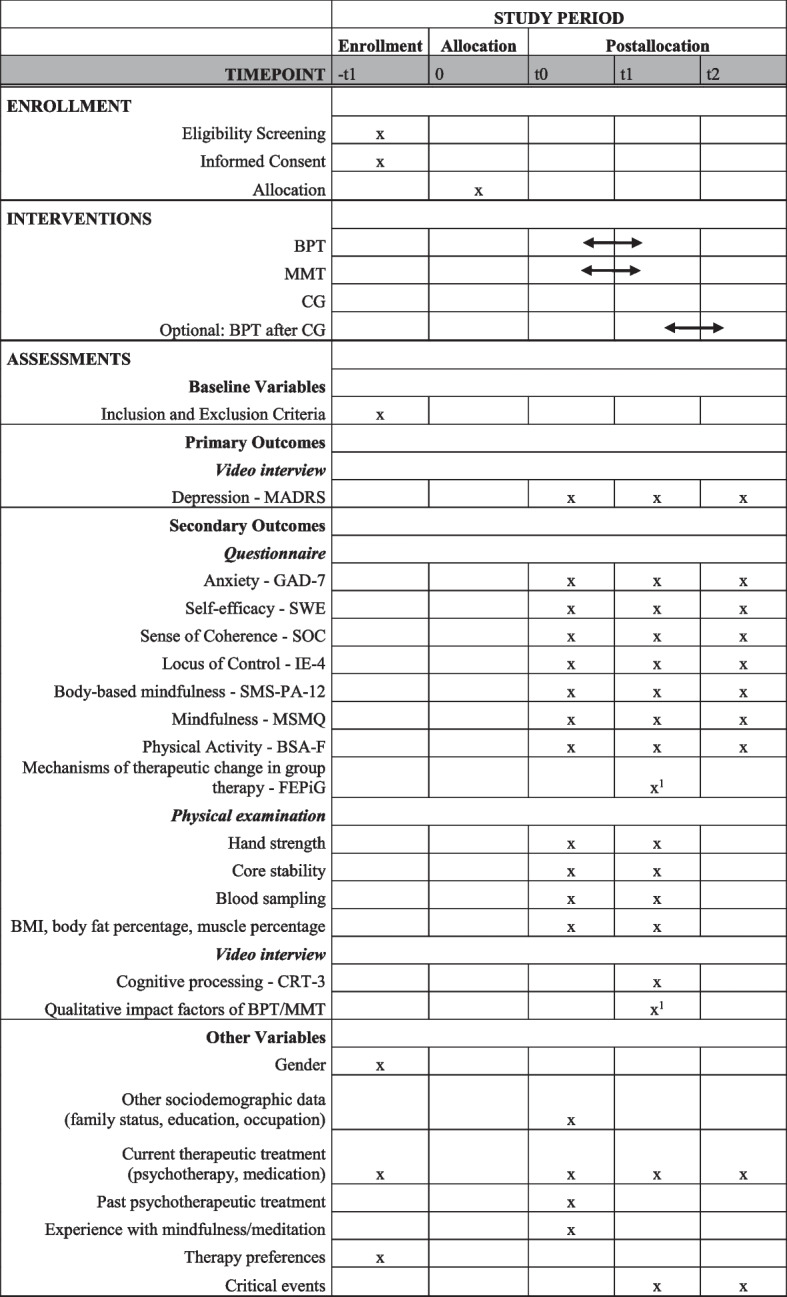
SPIRIT figure. Participant timeline. Superscript digit one (^1^) indicates the following: only participants in the intervention groups. BMI, body mass index; BPT, bouldering psychotherapy; BSA-F, Bewegungs- und Sportaktivität Fragebogen; CG, control group; CRT-3, Cognitive Reflection Test; FEPiG, Fragebogen zum Erleben von therapeutischen Prozessen in der Gruppe; GAD-7, Generalized Anxiety Disorder—Scale-7; IE-4, Internal External Locus of Control; MADRS, Montgomery and Asberg Depression Rating Scale; MMT, mental model therapy; MSMQ, Multidimensional State Mindfulness Questionnaire; SMS-PA-12, State Mindfulness Scale for Physical Activity; SOC, Sense of Coherence Scale; SWE, Skala zur Allgemeinen Selbstwirksamkeit

The study was designed as a randomized, controlled, interventional, prospective, longitudinal, assessor-blinded study with three arms (BPT, MMT, CG). The overall start date was on October 1, 2021. Recruitment began on March 29, 2022, and will continue through February 28, 2023, in the Erlangen/Nuremberg/Fuerth region, a metropolitan region of three cities. After passing a diagnostic screening interview, participants are randomly assigned to one of the IGs (BPT or MMT) or the CG. Both IGs consist of 10 weekly 2-h sessions with a maximum of 11 participants and are each led by two therapists. After the end of the intervention phase, participants in the CG will be offered preferential admission to an outpatient BPT group at University Hospital Erlangen. MMT will be directly evaluated in an RCT design because many of its single components are well-established and well-investigated cognitive distortions. As it is very unlikely that the novel long-term focus on transforming thinking errors into appropriate goal-oriented thinking and action could lead to significant side effects, a previous open trial was not deemed mandatory. In all four consecutive waves of the study, the interventions are taking place simultaneously. To collect the data, structured video interviews are being conducted at baseline before the onset of therapy (t0), after the intervention (t1), and 12 months after the end of the intervention (t2). In addition to the interviews, participants are required to complete a series of questionnaires online (at t0, t1, and t2) and to undergo a physical examination with blood sampling (at t0 and t1 at about the same time of day). All procedures were approved by the Friedrich-Alexander-Universität Erlangen-Nürnberg Ethics Committee (Ref. 21-332-B) on December 13, 2021. The SPIRIT participant timeline is shown in Fig. 1, and the SPIRIT checklist is provided as an Additional file [51]. Trial Registration Data are presented in Table 1 (ISRCTN12347878, Registration date: March 28, 2022, registered prospectively).

**Table 1:** Trial registration data

**Data category**	**Information**
1. Primary registry and trial identification number’	ISRCTN 12347878
2. Date of registration in primary registry	March 28, 2022
3. Secondary identifying numbers	-
4. Source(s) of monetary or material support	University Hospital Erlangen
5. Primary sponsor	University Hospital Erlangen
6. Secondary sponsor(s)	-
7. Contact for public queries	see Point 8
8. Contact for scientific queries	PD Dr. Katharina Luttenberger, katharina.luttenberger@uk-erlangen.de
9. Public title	New ways to cope with depression
10. Scientific title	New ways to cope with depression—study protocol for a randomized controlled mixed methods trial of bouldering psychotherapy (BPT) and mental model therapy (MMT)
11. Countries of recruitment	Germany
12. Health condition(s) or problem(s) studied	Depression
13. Intervention(s)	Intervention group receiving the bouldering psychotherapy (BPT)
	Intervention group receiving the mental model therapy (MMT)
	Control group (CG)
14. Key inclusion and exclusion criteria	Inclusion criteria: 1. Depressive episode, assessed via virtual diagnostic screening interview based on the DSM-V diagnostic criteria 2. Informed consent to participate in the study (especially regarding the randomized allocation, anonymous storage of data, and data collection) 3. Ability and availability to come to the therapy locations and time to participate as well as access to digital applications for video or telephone interviews
	Exclusion criteria: 1. Age < 18 years 2. Presence of current severe mental illnesses that complicate participation in group therapy (e.g., psychosis, mania, current self-injury, acute substance dependence, current suicidality) 3. Physical contraindication for climbing (physical ailments or pregnancy) 4. Body Mass Index (BMI) < 17.5 or > 40 5. Current treatment confounded with study outcomes (participation in psychotherapeutic group therapy, initiation/change in psychotropic medication within 8 weeks prior to the intervention)
15. Study type	Randomized controlled prospective assessor-blinded longitudinal clinical intervention study
16. Date of first enrollment	Began on March 29, 2022
17. Target sample size	120
18. Recruitment status	Ongoing
19. Primary outcome(s)	Depression measured with the Montgomery Asberg Rating Scale (MADRS) score from a third-party assessment in computer-assisted video interviews
20. Key secondary outcomes	Anxiety, self-efficacy, sense of coherence, locus of control, (mind- and body-based) state mindfulness, physical activity, mechanisms of therapeutic change in group therapy, blood concentration of osteocalcin, sphingolipid parameters, growth factors and inflammatory markers, qualitative factors of influence
BPT specific	Hand strength, core stability, BMI, body fat percentage, muscle percentage
MMT specific	Cognitive processing

### Sample size estimation

ITT sample size was calculated on the basis of the authors’ previous experiences with BPT. Both studies on the effectiveness of BPT showed a moderate effect size (Cohen’s *d*s of 0.55 to 0.77) on depression severity [24, 25]. On the basis of effect sizes for CBT [9], comparable effect sizes are expected for MMT. Thus, an a priori power analysis to identify a moderate effect of *f* ≥ 0.295 was computed with the statistical program G*Power version 3.1.9.7. The calculations for the mixed ANOVA with two repeated measures as within-subjects factors and three groups as between-subjects factors and a group × time interaction revealed a total sample size of 114 participants. Power was set at 80%, *α* = .05, a correlation between repeated measurements of .50, and a nonsphericity correction of 1. To create three equal-sized groups each consisting of 10 participants across the four waves of recruitment, we planned to recruit a total of 120 participants, resulting in a total of 40 participants per group.

### Recruitment strategies

We are using several recruitment strategies to ensure the highest possible representativity of the sample: Informational materials (e.g., flyers, posters) are being distributed throughout the Erlangen/Nuremberg/Fuerth region at psychiatric hospitals, psychotherapist offices, primary care physician offices, pharmacies, health insurance companies, and other psychological and social services (e.g., support groups, counseling centers). Information is also being shared with psychotherapists, psychiatrists, and primary care physicians in outpatient clinics, outpatient training clinics, and private practices, asking them to pass the offer on to patients on their waiting lists. People who have expressed interest in participating and have agreed to be contacted for the upcoming or a later wave of the study are being contacted via email. Before each wave, press releases are being issued and addressed to a variety of local newspapers, and general informative events are being held in auditoriums at the University Hospital Erlangen and online via the platform *Zoom Meetings*. On the Internet, a homepage [52], an Instagram account, and a Facebook account were created and are being updated regularly with the most current information.

### Eligibility of participants

All people interested in participation are invited to attend one of the three general informative events held by the study personnel in each wave. Relevant information about the IGs and an explanation of the participation conditions (e.g., randomization procedure please see “Randomization”) are provided there. Individuals who decide to participate are asked to undergo a computer-assisted diagnostic video screening interview for inclusion and exclusion criteria and to provide written informed consent.

#### Inclusion and exclusion criteria

To increase external validity, only a few inclusion and exclusion criteria are applied. *Inclusion criteria* are fulfilling the diagnostic criteria for an episode of major depression (single or recurrent) according to DSM 5, informed consent to participate in the study (especially regarding randomized allocation and data acquisition), and the ability and time to travel to the therapy locations to participate in the interventions, as well as access to digital applications for video or telephone interviews. The presence (or absence) of depression is assessed by a standardized diagnostic screening interview that is based on the Diagnostic and Statistical Manual of Mental Disorders, Fifth Edition (DSM-V), diagnostic criteria [53]. *Exclusion criteria* are an age younger than 18 years, a body mass index (BMI) under 17.5 or over 40 (for safety reasons in BPT), current participation in another psychotherapeutic group therapy, an initiation of a change or a change in psychiatric medication 8 weeks (or fewer) before the study (starting a medication more than 8 weeks before the study is not a reason for exclusion), a planned inpatient stay during the intervention period, physical contraindication for bouldering (physical ailments or pregnancy), specific severe psychiatric disorders, and acute suicidality. Severe psychiatric disorders leading to exclusion from the study are psychosis within the last 5 years, a manic episode within the last 5 years, substance addiction with substance abuse within the last year, self-harming behavior during the last year, and current organic diseases with symptoms of psychiatric disorders, such as dementia or delirium. For safety reasons, all applicants are screened for suicidality, and only those who are able to comply with a safety plan and are in control of their own safety are included in the study. The safety plan, which is discussed with the applicants, includes information about in-patient crisis intervention at the nearby psychiatric hospital and the 24-h crisis intervention telephone hotline provided by the local mental health services. All inclusion and exclusion criteria are assessed in a diagnostic screening interview by the study personnel.

### Randomization

Blockwise randomization per study wave was performed externally by the Institute of Medical Informatics, Biometry, and Epidemiology (IMBE), Friedrich-Alexander-Universität Erlangen-Nürnberg, only sharing patients’ code, age, sex, and treatment preference. The latter three were used in a stochastic minimization algorithm to reduce group imbalance [54]. More specifically, in each wave, 1000 random permutations of group memberships for the new patients (with equally sized groups) were generated, and the first permutation that minimized the summed chi-square or *F* statistic for group differences with respect to sex, treatment preference, and age was selected; the statistician transmitted the sampled group allocations back to the study center. The randomization algorithm was implemented in the statistical software environment R [55]. After the randomization, participants are informed about their allocation (BPT, MMT, CG) and are given detailed information about their group participation.

#### Masking

Whereas participants are informed about which treatment condition they are allocated to, the interviewers conducting the video interviews and thus assessing the outcomes of the study are blind to the participants’ group allocations. At the beginning of each interview, the participant is reminded to remember the confidential nature of their allocation and not to tell the interviewer which treatment they are receiving.

### Interventions

#### Bouldering psychotherapy (BPT)

The bouldering intervention is a combination of bouldering and psychotherapy and consists of 10 consecutive sessions. Unlike rock climbing, bouldering is defined as climbing to moderate heights (up to about 3 m) without the use of ropes or harnesses. Potential falls or jumps are protected by thick mats. The wide range of difficulty levels (usually indicated by different colors) makes it possible for people with different levels of fitness to boulder together in the same group. The sessions are 2 h each, taking place in a bouldering gym in Erlangen every week in the late afternoon. Each group consists of approximately 10 participants who are supervised by a team of two climbing therapists. All of the three climbing therapists are psychotherapists with bouldering and rock-climbing experience, and at least one member of the team has received advanced training form the German Alpine association, including safety requirements in bouldering.

The contextual focus of each session is on a specific psychological topic considered to be relevant in the development and maintenance of depression (see Table 2).

**Table 2:** Overview of the bouldering therapy sessions

**Session**	**Topic**
1	Introduction to bouldering and mindfulness
2	Body awareness and the body’s center of gravity
3	Dealing with boundaries
4	Expectations and aspirations
5	Self-efficacy, achievement, and pride
6	Self-worth
7	Fear and confidence I
8	Fear and confidence II
9	Social relationships
10	Closing: problem solving, reflection on lessons learned, transfer to everyday life, and farewell

In the BPT sessions, therapists follow a BPT manual [56] that includes a fixed schedule for each session to provide a standardized implementation of the BPT (www.bouldering-psychotherapy.com). All of the 10 sessions consist of three main parts: introduction (about 20 min), action phase (about 75 min), and closing (about 25 min). The introduction phase is held in a room that is separate from the bouldering hall (e.g., the yoga room) and begins with a mindfulness exercise (e.g., breathing meditation) to help participants settle in and focus their attention on the present moment. The exercise is followed by a presentation of the session’s topic and psychoeducation (e.g., function and body signals of anxiety). At the end of introduction phase, participants engage in a brief exchange about their experiences with the topic. Afterwards, the group is divided into two smaller groups that are each supervised by one therapist from that point on. During the action phase, participants complete one or two topic-related bouldering exercises that are supposed to evoke underlying emotions (e.g., anxiety), uncover participants’ characteristic patterns (e.g., avoidance), and enable them to engage in new experiences (e.g., exposition: bouldering blindfolded) with the support of the therapists. This phase takes place in the bouldering hall. After completing these exercises, the remaining time is used for free bouldering during which everyone works on their individual “projects” while being supported by the therapists. For the closing phase, the two groups reunite in the enclosed room, talk about their experiences during the bouldering exercises, and develop ideas about how to integrate the lessons they learned into their everyday lives. All sessions end with a body-related relaxation exercise.

#### Mental model therapy (MMT)

Mental model therapy is based on the assumption that internalized cognitive distortions, biased decision making, and false assumptions lead to stress and reduced self-esteem. The goal of MMT is to trace these cognitive errors and replace them with more useful mental models (see Table 3). The central mental model is the Eisenhower principle, which helps prioritize what is really important: By distinguishing between important versus less important activities crossed with urgent versus not urgent activities (four quadrants), participants learn to overcome the natural tendency to focus on unimportant urgent tasks and instead learn to engage in long-term activities that are crucial for their well-being. MMT is expected to reduce stress and depressive symptoms and to have a preventive effect on future mental disorders by promoting a sense of coherence, resilience, and resources. The intervention consists of 10 consecutive, weekly 2-h sessions that take place in groups of about 10 participants in the psychiatric unit at the University Hospital Erlangen. Each group is led by a team of two therapists (out of three different therapists) who were specially trained in manualized MMT before the intervention. Equivalent to the qualification of the BPT therapists, all MMT therapists are psychiatrists, psychotherapists, or psychologists who are in the process of completing their psychotherapy hours (according to German law, psychotherapists must have an MSc degree in psychology plus an additional 4200 h of additional education as a psychotherapist). All therapists are experienced in the treatment of depression. Comparable to the BPT intervention, each session focuses on a specific psychological topic concerning cognitive distortions and misconceptions (see Table 3).

**Table 3:** Overview of the MMT sessions

**Session**	**Topic**
1	Introduction, Eisenhower principle
2	Habits & Pareto principle
3	Balance model & Ivy-Lee Method
4	Solomon paradox [57] & Focusing illusion
5	Saying no & Mental models
6	Active language & Confirmation bias [58]
7	Compass of life & Sunk cost fallacy [59]
8	Hindsight bias [60] & Decisions
9	Radical acceptance [61] & Occam’s razor [62]
10	Closing: Reflection and transfer to everyday life, farewell

In the MMT sessions, therapists follow a manual that describes how to structure each session to ensure a standardized implementation. All 10 sessions consist of the following phases: a starting phase (about 20 min), one or two active phases with a break of 10 min (about 85 min), and a closing phase (about 15 min). The starting phase begins with a brief round in which everyone shares their current state of mind, followed by a discussion about the last session’s homework and difficulties that may have occurred. In order to create a more personal atmosphere and to give everyone the opportunity to exchange ideas, the participants will be divided into smaller groups with rotating members for the first phase. During the active phase(s), the small groups are reunited so that they can provide input on the sessions’ topics before they are divided into smaller groups again for a topic-related intervention. The intervention is followed by a discussion in the whole group again about participants’ experiences and difficulties. Some interventions do not require teamwork and are carried out as individual work. After the active phase(s), the session ends with a closing phase consisting of a homework assignment and everyone sharing their take-home message.

#### Control group (CG)

The CG receives no study-specific additional therapy during the intervention phase but is free to undergo all established psychotherapeutic and psychiatric treatment options and therefor represent treatment as usual in the German Health Care System. Participants allocated to the CG are given city vouchers (that can be redeemed in numerous regional shops and locations) or free passes to the local bouldering hall after completing measurement times t1 and t2. If interested, they are also offered preferential admission to the outpatient BPT group at Erlangen University Hospital after t1.

### Data collection

The target variables are being collected with several self-report questionnaires, standardized videoconference interviews, and physical examinations. The secure patient software *Samedi*, which is certified by the German Association of Statutory Health Insurance Physicians (KBV) (DSGVO-compliant), is being used to schedule interviews and examinations as well as to conduct the video interviews. All data are being collected with the web-based data collection system *REDCap2*. Each group is examined before the intervention (t0), again at the end of the 10-week intervention period (t1), and for a third time about 12 months after the end of the intervention (t2). Participants are instructed to answer the questionnaires before their scheduled appointments. The video interviewers are students who are studying either clinical psychology or medicine and have undergone training at the study’s headquarters. The physical examinations, including the blood sampling, are performed before the therapy begins (t0) and again after it ends (t1) by trained students of medicine only.

### Measures

All measures used over the course of the study at the different measurement points are listed in Fig. 1.

#### Primary outcome measures

##### Montgomery-Asberg Depression Rating Scale (MADRS) [63]

The MADRS is one of the most commonly used rating scales for assessing the core symptoms of depression [64]. It is conducted as a structured clinician-rated interview consisting of 10 items: apparent sadness, reported sadness, inner tension, reduced sleep, reduced appetite, concentration difficulties, lassitude, inability to feel, pessimistic thoughts, and suicidal thoughts. Each item is rated on a 7-point scale ranging from 0 to 6 with higher scores indicating greater severity of symptoms. A score greater than 31 on the MADRS indicates severe depression, whereas a score of 10 or below indicates remission [65, 66]. Originally, the scale was published without wording suggestions for clinicians to help them collect the information required to rate the items. For this reason, we used the *structured interview guide for the Montgomery-Asberg Depression Rating Scale (SIGMA)* [64]. The SIGMA is a structured interview guide that offers a selection of different questions for each of the 10 items with good to excellent interrater reliabilities [64].

#### Secondary outcome measures

##### Generalized Anxiety Disorder Scale-7 (GAD-7) [67]

The GAD-7 is used to assess anxiety. It is a short self-report questionnaire with seven items for assessing how often participants have experienced the seven core symptoms of generalized anxiety disorder during the last 2 weeks. Items are rated on a 4-point scale ranging from 0 (*not at all*) to 3 (*nearly every day*). Total scores can range from 0 to 21, with scores ≥ 5 indicating mild anxiety symptoms, scores ≥ 10 moderate anxiety symptoms, and scores ≥ 15 severe anxiety symptoms [67].

#### Skala zur Allgemeinen Selbstwirksamkeit (SWE) [68, 69]

The SWE is used to measure self-efficacy, which refers to the subjective belief that one is able to cope successfully with difficult situations. The questionnaire consists of 10 items that are answered on a unidimensional 4-point scale ranging from 1 (*not true*) to 4 (*exactly true*). Individual test scores can range from 10 to 40, with higher scores indicating higher self-efficacy [68, 69].

#### Sense of Coherence scale (SOC) [70, 71]

The short nine-item version of the SOC (SOC-L9) is used to measure participants’ sense of coherence, which is defined as a global belief concerning the extent to which individuals have a general enduring and dynamic sense of confidence that they are able to make reasonable and probable predictions about developments in their internal and external environment [70]. The nine items are rated on a unidimensional 7-point scale ranging from 1 to 7 with different verbal labels at each end (e.g., frequency, agreement). Scores range from 9 to 63, with higher scores implying a higher sense of coherence [70, 71].

#### Internal-External Locus of Control Scale (IE-4) [72]

The IE-4-scale is used to measure locus of control, containing a subscale for internal locus of control and a subscale for external locus of control with two items each. An internal control belief is associated with the extent to which a person is convinced that they are able to control events, whereas an external control belief refers to the extent to which a person believes that events and experiences are left to fate or chance or are controlled by other people over whom they have no control [72]. The items are rated on a 5-point response scale ranging from 1 (*not at all true*) to 5 (*completely true*). The scores are averaged for each subscale, resulting in scores that range from 1 to 5. Higher scores indicate higher internal or external locus of control [72].

#### State Mindfulness Scale for Physical Activity (SMS-PA-12) [73, 74]

The SMS-PA-12 is used to measure state mindfulness of the mind and of the body. The first refers to the concept of self-regulation of attention to being present without judgment as well as openness to, curiosity about, and acceptance of mental and physical experiences, whereas the latter describes how much attention people pay to their physical effort or bodily engagement. The SMS-PA-12 consists of 12 items with the subscales mindfulness of the mind and mindfulness of the body, each of which contain six items. All items are rated on a 5-point scale ranging from 0 (*not at all*) to 4 (*very much*). The SMS-PA-12 supports the use of a single score for overall state mindfulness as well as two separate scores for the subscales and has up to now not been validated in the German language [73, 74].

#### Multidimensional State Mindfulness Questionnaire (MSMQ) [75]

The MSMQ is used to assess state mindfulness. Following a factor analysis, state mindfulness in the MSMQ can be viewed as the within-person component of mindfulness, contrary to trait mindfulness, which is described as the between-person component. Fifteen items are rated on a 7-point scale ranging from 0 (*does not apply at all*) to 6 (*applies completely*). The items provide information about three distinguishable facets of mindfulness: acting with awareness, nonjudgmental acceptance, and present-moment attention [75].

#### Physical Activity, Exercise, and Sport Questionnaire (Bewegungs- und Sportaktivität Fragebogen; BSA-F) [76]

The BSA-F is used to record the frequency, duration, intensity, and type of physical activities that participants engage in with respect to movement and sports. The scale is divided into three subscales that measure activities involving movement at work, activities involving movement during free time, and sports activity. Only the first and third subscales are included in the current study. Items measuring movement are answered on a 4-point scale ranging from 0 (*none*) to 3 (*a lot*). Sports activity is assessed with an open question about the type, frequency, and duration of sports and exercise that participants have engage in over the past 4 weeks. The data will be summarized in an index of sports activity [76].

#### Fragebogen zum Erleben von therapeutischen Prozessen in der Gruppe (FEPiG) [77]

The FEPiG is deployed to detect the factors of influence of the group therapies by assessing the experience of the therapeutic processes and the acceptance of the form of therapy. In the current study, only 30 of the 42 items are being used with an additional question about whether the participant would recommend the specific group therapy. The items are answered on a 5-point scale ranging from 0 (*not at all true*) to 4 (*completely true*). Assessable factors of influence are interpersonal learning, group cohesion, goals and tasks, catharsis, bonding with therapists, and problem actualization [77].

#### Baseline® hydraulic hand dynamometer [78]

The hydraulic dynamometer is deployed to measure hand or grip strength, which has been demonstrated to be a valid measure of muscle strength in general [79]. The handheld device is used with the left and right hands consecutively, and participants are instructed to squeeze with maximum force [78]. The values for each hand are compared with a sex- and age-specific norm table [80, 81].

#### McGill’s torso muscular endurance test battery [82]

The torso muscular endurance test battery measures core stability. It consists of the following three subtests: trunk flexor endurance test, trunk lateral endurance test, and trunk extensor endurance test. The goal of each exercise is to hold the required position for as long as possible while the time is measured with a stopwatch. The results are presented as three different ratios that can be rated as either good or poor: flexion to extension ratio, right-side bridge to left-side bridge ratio, and side bridge (each side) to extension ratio [82].

#### Analysis of blood biomarkers

Blood samples are being collected in 9-ml-serum and 9-ml-EDTA-plasma vials at both t0 and t1, centrifuged, and stored as serum and plasma aliquots, respectively, at – 80 °C. After lysis or erythrocytes, pellets of peripheral blood mononuclear cells will be frozen dry or stabilized with RNA later. The blood samples will be analyzed for the concentration of biomarkers, such as osteocalcin, growth and immune factors using commercial antibody-based ELISAs, and other detection methods. Enzyme activities of sphingolipid metabolism will be determined by established assays using fluorescently-labeled substrates [83]. Gene expression analysis will be performed by quantitative real-time PCR.

#### Body fat scale

We are using the personal scale to measure participants’ BMI, body fat percentage, and muscle percentage during the physical examinations.

#### Cognitive Reflection Test (CRT-3) [84, 85]

The CRT-3 is used to assess cognitive processing and performance on heuristics-and-biases tasks. It measures the tendency to engage in the kind of reflection that leads to the correct answer instead of choosing the prepotent but incorrect response alternative. The test consists of three questions [84, 85].

#### Qualitative interviews

In computer-assisted qualitative interviews, Grawe’s factors of influence (e.g., resource activation, problem actualization, motivational clarification, problem solving, and therapeutic relationship) [86] are being assessed for both IGs. The interviews are based on guidelines for semi-structured interviews that are being conducted to collect additional potential factors of influence that go beyond the previously mentioned concepts: previous experiences with (group) therapies; individual changes throughout the intervention; feelings related to the group, the therapists, and the therapy itself; negative experiences or effects; and potential barriers. In accordance with Mayring’s [87] specifications, a qualitative content analysis that follows the principles of thematic analysis [88] will be conducted to evaluate all qualitative data.

#### Other variables

During enrollment, interested people are participating in a computer-assisted diagnostic video *screening interview* to assess the inclusion and exclusion criteria before being told whether they can participate in the study. The interviewer assesses the following data: *age*, *sex*, *height*, and *weight* to determine *body mass index* (BMI), *current outpatient group therapy*, *current psycho-pharmacotherapeutic treatment*, *physical limitations*, *psychiatric comorbidities*, *preferences concerning future group allocation* (BPT, MMT, CG), and *depressive symptoms*. The presence of a current depressive episode is measured with a structured interview based on the DSM-V diagnostic criteria [53]. It includes the assessment of the participants’ current depression as well as past episodes and age of onset.

At baseline, further sociodemographic data, such as *family status*, *level of education*, and *current occupation*, are being assessed via a questionnaire. In a video interview, the following additional data are being collected: *current and past psychotherapeutic treatment* (inpatient and outpatient), *experiences with mindfulness and meditation*, and *participation in sports for the treatment of depression*.

*Changes concerning current psycho-pharmacotherapeutic and psychotherapeutic treatment* are being assessed with a video interview shortly after as well as 12 months after the intervention.

*Critical events*, such as injuries, accidents, mental crises, or hospitalization, as well as their intensity (mild, medium, severe), during the intervention period and during the year following the intervention are being assessed shortly after and 12 months after the intervention. The intensity of these events is judged on the basis of the extent of the impact on the participant’s life and the resulting need for treatment.

### Data quality management and data protection

The student interviewers have been thoroughly trained and are being supervised by the study center’s staff. If the interviewers have questions, they can contact the study center’s staff for the duration of the study period. The therapists of both IGs constantly remain in close contact with the study center and discuss all uncertainties and therapeutic progress in regular appointments and intervisions among their colleagues in MMT or BPT from the study headquarter. Regular attendance at therapies and potential adverse events are documented on a weekly basis during the intervention periods. Adverse events are classified by severity into mild, moderate, and severe adverse events. Severe adverse events (SAEs) include, for example, severe head injury, spinal cord injury, mental health crisis resulting in hospitalization, suicide attempt, and death.

To test for interrater reliability, 10% of both the pre- (t0) and post- (t1) MADRS surveys as well as 10% of the qualitative interviews will be rated by two differently paired raters. The quality of the data will be guaranteed by strict data monitoring at the study center over the entire study period.

Data handling is in line with European and German data protection laws, and the information sheet contains a data protection declaration. All sensitive data gathered during the video interviews and physical examinations is being pseudonymized and stored in password-protected devices and programs. The participants’ names, contact information, and corresponding codes are being collected in a separate password-protected document. Only members of the study team have access to this list. Patient information sheets are being digitalized and stored in password-protected devices. Published material will not contain any patient-identifying information. The blood samples taken during the physical examination are either being used up or the residues will be destroyed after the analyses are completed.

### Data analysis

The *IBM SPSS Statistics V.28* software will be used to analyze the data. All relevant primary and secondary outcome variables as well as sociodemographic data will be reported descriptively. Participants who drop out over the course of the study but are still available for the video interviews will still be interviewed. After a missing data evaluation is carried out, missing outcome data will be imputed by EM-Imputation [89]. The primary data analytic strategy will be intention to treat (ITT). As a sensitivity analysis additionally, a per-protocol (PP) analysis will be performed. The significance level will be set at *α* = .05.

#### Quantitative measurements

The primary hypothesis will be tested via mixed model ANOVAs with which potential interactions between time and group can be detected. To test the first and second hypotheses, a mixed model ANOVA with the between-subjects factor group and the within-subjects factor time as well as the group × time interaction will be calculated. Additionally, multiple regression analyses will be calculated to control for the effects of possible confounding variables (e.g., demographics, additional therapeutic treatments, such as antidepressant medication and psychotherapy).

Secondary outcome variables will be tested in an exploratory fashion with mixed model ANOVAs.

#### Qualitative data

The qualitative data on the factors of influence of the IGs will be analyzed using the software *MAXQDA*. Qualitative content analysis will be performed in accordance with Mayring’s specifications and the principles of thematic content analysis [87, 88]. First, a literature-based coding system encompassing the known factors of influence of psychotherapy will be developed [77]. This approach will be informed from the bottom-up development of codes from the interviews. The coding system will be developed by at least two trained researchers and its aptness verified in an iterative process according to the material. Around 10% of the interviews in each group will be used to calculate the interobserver agreement.

## Discussion

In this article, we presented the design for a randomized controlled trial to test the efficacy of two short-term group therapies for outpatients with depression using a mixed methods approach. In addition to quantitative data collection, qualitative data on factors of influence for each intervention will be analyzed. The study could offer important insight into the effectiveness and potential factors of influence of short-term group therapies that have up to now been less well studied than individual therapies [13].

The group therapies that are being investigated in the present study are, first, BPT, whose effectiveness has already been investigated in two RCTs [23, 25] and, second, a newly developed cognitive-based group therapy with mental models.

Both interventions consist of innovative new elements with an effective therapeutic background: BPT includes contents of physical activity and body experience, MMT the element of mental models with a long-term influence derived from research on decision making [30, 31]. As a significant number of patients still do not profit from existing psychotherapies [10], it seems sensible to develop different types of therapies and identify possible factors of influence in order to be able to personalize the therapeutic technique to enhance the benefits of this technique for all at a later stage of development.

During and after the intervention period, participants are free to begin or continue other therapeutic treatments offered by the German health care system without suffering any disadvantages. Previous ongoing treatment plans will not be changed by their participation in this study. Therapeutic treatment outside of the study will be assessed in the video interviews.

Participation in the BPT is accompanied by a slight risk of injuries; however, this level of risk does not exceed the risks of other physical activities. In a study on sport-climbing injuries, an average number of 0.2 injuries per 1000 h of climbing (which has higher risks of severe injuries than bouldering) occurred in a sample of about 2000 climbers [90]. In our study, bouldering time is limited to a maximum of 10 × 2 h per patient. Hence, severe injuries are not expected. Irrespective of their group allocation, all participants are provided with road and accident insurance while participating in the study. In our previous studies, no SAEs occurred in more than 4000 h of bouldering. Nevertheless, interim analyses of SAEs are being performed on a regular basis in both intervention groups. If any analyses show a cumulation of SAEs in one group, the respective intervention will be terminated.

All participants’ suicidality and capability of collusion are being assessed during the diagnostic screening interview and at all measurement points in the video interviews as part of the MADRS. During the therapy sessions, therapists follow good clinical practice in monitoring for suicide risk. In cases of acute risks, they will establish suicide risk management plans.

The strengths of the present study are the randomization, the mixed methods approach, the naturalistic design with very few exclusion criteria, and the longitudinal character of the study with a long follow-up period of 1 year after the end of treatment. The use of manualized treatments in a naturalistic setting will contribute to the high internal and external validity of the study. The inclusion and exclusion criteria are being assessed via diagnostic screening interviews in contrast to self-report assessments. Additionally, the severity of depressive symptomatology as the primary outcome is being collected by blinded interviewers, and the interrater reliability is being assessed. A methodological limitation might be a possible selection bias due to people choosing to participate because they are interested in the therapies. Therefore, group preference is being taken into account during randomization, and it will be possible to compare subgroups with respect to their group preference. Also, the selection of a specific therapy on the basis of interest in the intervention is naturalistic and will hence contribute to higher external validity. Another limitation is the fact that the psychotherapeutic interventions themselves cannot be blinded. Furthermore, the clinical heterogeneity of the sample could lead to a reduction in effect sizes but would also strengthen external validity. As participants are free to continue with or begin other individual treatments during the study, possible confounding effects due to simultaneous interventions cannot be ruled out. However, ongoing and new treatments as well as changes in treatment plans during the study will be assessed at all measurement points, and the data will be considered in the analyses.

### Supplementary Information

**Additional file 1.** SPIRIT Checklist for Trials.

## Data Availability

The research group intends to publish data generated from this study in high-impact peer-reviewed open-access journals. Data are stored on protected devices at the University Hospital Erlangen. Fully anonymized data will be available upon reasonable request after the publication of results. The trial registry will be updated if protocol modifications are made. Model consent forms in the German language were approved by the Ethics Committee and will be made accessible upon request along with the participant information materials.

## Abbreviations

BMI: Body mass index
BPT: Bouldering psychotherapy
BSA-F: Bewegungs- und Sportaktivität Fragebogen
CBT: Cognitive behavioral therapy
CG: Control group
CRT-3: Cognitive Reflection Test
DSM-V: Diagnostic and Statistical Manual of Mental Disorders, Fifth Edition
FEPiG: Fragebogen zum Erleben von therapeutischen Prozessen in der Gruppe
GAD-7: Generalized Anxiety Disorder Scale-7
IE-4: Internal External Locus of Control
IGs: Intervention groups
ITT: Intention to treat
MADRS: Montgomery and Asberg Depression Rating Scale
MMT: Mental model therapy
MSMQ: Multidimensional State Mindfulness Questionnaire
PP: Per protocol
SAEs: Severe adverse events
SMS-PA-12: State Mindfulness Scale for Physical Activity
SOC: Sense of Coherence Scale
SWE: Skala zur Allgemeinen Selbstwirksamkeit
